# Electron Beam Melting of Niobium Alloys from Blended Powders †

**DOI:** 10.3390/ma14195536

**Published:** 2021-09-24

**Authors:** Jameson P. Hankwitz, Christopher Ledford, Christopher Rock, Scott O’Dell, Timothy J. Horn

**Affiliations:** 1 Department of Materials Science and Engineering, North Carolina State University, Raleigh, NC 27695, USA; jphankwi@ncsu.edu; 2 Manufacturing Science Division, Oak Ridge National Laboratory, Oak Ridge, TN 37830, USA; ledfordcc@ornl.gov; 3 Center for Additive Manufacturing and Logistics, North Carolina State University, Raleigh, NC 27695, USA; cdrock@ncsu.edu; 4 Plasma Processes, Huntsville, AL 35811, USA; 5 Department of Mechanical and Aerospace Engineering, North Carolina State University, Raleigh, NC 27695, USA

**Keywords:** niobium, tungsten, zirconium, high-strength metal alloys, plasma spheroidization, additive manufacturing

## Abstract

Niobium-based tungsten alloys are desirable for high-temperature structural applications yet are restricted in practice by limited room-temperature ductility and fabricability. Powder bed fusion additive manufacturing is one technology that could be leveraged to process alloys with limited ductility, without the need for pre-alloying. A custom electron beam powder bed fusion machine was used to demonstrate the processability of blended Nb-1Zr, Nb-10W-1Zr-0.1C, and Nb-20W-1Zr-0.1C powders, with resulting solid optical densities of 99+%. Ultimately, post-processing heat treatments were required to increase tungsten diffusion in niobium, as well as to attain satisfactory mechanical properties.

## 1. Introduction

Niobium (Nb)-based refractory alloys are of significant interest for high-performance components in extreme environments, such as the STAR thruster [1] and leading edge heat pipes for aerospace vehicles [2]. In its pure state, Nb does not possess sufficiently high mechanical properties at elevated temperatures (>1000 °C) for these applications. This includes cold-worked Nb, which achieves greater tensile strengths than annealed Nb, up to 585 MPa [3], but does not retain increased strengths at high temperatures (600 °C, according to Barlett et al.) [4,5]. Additionally, Nb readily oxidizes above 400 °C—at which point, oxygen embrittlement and large residual stress formation occur, resulting in reduced creep strength [6,7].

Alloys have been developed to improve the strength retention of pure Nb at high temperatures that leverage precipitate-forming constituents such as hafnium (Hf), zirconium (Zr), and titanium (Ti), that react with oxygen or carbon during processing to form high-melting-temperature oxides and carbides. These precipitates pin the grain boundaries and prevent excessive grain growth at elevated temperatures. Additionally, the high-temperature strength of Nb is further improved by elements in solution such as tungsten (W), molybdenum (Mo), and tantalum (Ta) [8,9,10].

The tradeoff for this increase in high-temperature strength is a reduction in room-temperature ductility and formability. Three categories for Nb alloys have previously been defined; low strength with high ductility, moderate strength and ductility, and high strength with low ductility [8,9,11]. Alloys that populate these categories include Nb-1Zr and PWC-11, D-43, and AS-30, respectively. PWC-11 (Nb-1Zr-0.1C) is ZrC strengthened with substantial ductility, which has allowed both Nb-1Zr and PWC-11 to be commercially used and researched [4,12,13]. However, their tensile strengths degrade quickly over 1200 °C, and neither has sufficient creep strength above ~900 °C [14]. D-43 (Nb-10W-1Zr-0.1C) and AS-30 (Nb-20W-1Zr-0.1C) are two alloys that combine solid solution and precipitation strengthening mechanisms. It has been observed that 20–30 wt% W additions are necessary in order to retain high strengths at 1400 °C [15]. However, the W-containing alloys have relatively limited ductilities, rendering them difficult to fabricate via forming or casting methods [11,16]. Consequently, while the promising nature of this class of materials has been well established, their degree of commercial implementation varies considerably based on the merit of their formability, weldability, and oxidation resistance [6,13].

Additive manufacturing (AM) is a processing route that has the potential to circumvent limitations of formability imparted by W additions. Application of AM to Nb has been predominantly focused on pure Nb [17,18,19,20,21,22]. Currently, the literature pertaining to EB-PBF of other non-formable refractory alloys is limited; Mo+TiC [12], WC-Co [23], and Nb-TiAl [24] are a few examples. Though these efforts have yielded various degrees of success in terms of density, cracking, and alloy homogenization, they collectively suggest that non-fabricable high-strength Nb alloys can also be processed by EB-PBF. A recent publication by Phillips et al. demonstrated AM of C-103 (Nb-10Hf-1Ti) components through both laser powder bed fusion (L-PBF) and electron beam powder bed fusion (EB-PBF) [25]. In their study, EB-PBF resulted in highly dense C-103 with low oxidation. This was attributed to the high ambient temperature and the vacuum environment of EB-PBF. The grain structure exhibited the columnar microstructure typical of AM and room-temperature strength was slightly lower than cast-wrought C-103, while the elevated-temperature strength was comparable to cast-wrought C-103. For both the room-temperature and elevated-temperature tests, the yield strength and elongation were above the minimum specification requirement for C-103 [22]. While C-103 is considered formable, these data suggest that a similar methodology could be applied to a family of Nb alloys predominately strengthened by additions of tungsten which have greater strength at high temperature than C-103.

Blended powder feedstocks for EB-PBF can expedite material development and reduce preparation costs, but interdiffusion between constituents remains a challenge [26,27,28]. Previous work involving L-PBF blended refractory powders with large melting point gaps have been published recently [29,30]. These studies suggest that there is a narrow processing window for achieving a fully solutionized microstructure (high energy requirement) without inducing keyhole porosity (low energy requirement). Of the different types of blended powder, chemically processed powder is the most cost-effective option. Chemical processing is usually followed by a crushing step which produces angular powder. Angular powder is not preferable for EB-PBF due to spreadability issues; however, it can be post-processed through plasma spheroidization to partially remelt and smoothen the powder. Furthermore, spheroidization can be used to alloy blended, chemically dissimilar powder, which circumvents atomization methods that are either expensive or otherwise incompatible with reactive refractory metals such as Nb and W.

In this study, we explore the feasibility of utilizing blended powders in EB-PBF for the fabrication of Nb alloys with a range of W content (0–20 wt%); Nb-1Zr, Nb-10W-1Zr-0.1C, and Nb-20W-1Zr-0.1C. Powders were produced by hydrogenation-dehydrogenation (HDH), crushing, spheroidization, and blending. Process parameters yielding high-density structures were identified and facilitated tensile bar fabrication to gauge mechanical properties. Heat treatments were also conducted with the intent to further solutionize and improve the mechanical properties of the samples.

## 2. Materials and Methods

### 2.1. Powder Feedstock Preparation

Three powder feedstocks were produced for this study. The base alloy, Nb-1Zr, was formed by hydride-dehydride (HDH) precursor material and screened in an argon glovebox to a nominal −140/+325 mesh fraction (45–106 µm). W powder was screened to −325/+625 mesh (15–45 µm) before blending with approximately 5 kg of the Nb-1Zr powder and carbon black particles (<1 µm) by 3D mixing for 2 h using a Turbula mixer (Glen Mills, Clifton, NJ, USA) and W milling media. Mechanical alloying was intentionally avoided to prevent fine powder formation. 10 wt% W and 0.1 wt% C were added to reflect D-43 nominal levels, while 20 wt% W and 0.1 wt% C were added to form AS-30. All powder mixtures were spheroidized using the Powder Alloying and Spheroidization (PAS) process [31]. During PAS processing, powder feedstock is fed through a plasma flame, capable of reaching 10,000 K, where both spheroidization and alloying can occur depending on particle size, melting point, degree of contact between constituent particles, and residence time in the plasma flame. To minimize oxidation of feedstock during processing, the chamber was evacuated twice and high purity argon was used as the shielding gas.

The powder size distribution was determined by laser diffraction using a Microtrac S3500 analyzer (Microtrac Inc., Montgomeryville, PA, USA) with ultrasonic agitation. The composition of the powder and solids was determined by a combination of inductively coupled plasma mass spectroscopy (ICP-MS, LECO OH836, LECO, St. Joseph, MI, USA) and inert gas fusion . A qualitative assessment of powder morphology was performed using a JOEL 6010LA (JEOL USA, Peabody, MA, USA) scanning electron microscope (SEM) in backscatter composition mode (BEC) with electron dispersive spectroscopy (EDS).

### 2.2. Sample Fabrication

All samples were fabricated using a customized Arcam S12 EB-PBF machine (GE Additive, Cincinnati, OH, USA) (software version 3.2). As described in our previous work [32,33] the build platform was modified to contain a reduced size (88 mm diameter × 35 mm) CP Ti substrate. Process monitoring was performed with standard machine thermocouples (type K) located below the build plate, a 2-color pyrometer (Fluke Process Endurance Pyrometer) focused on the center of the powder bed, and an IR camera (FLIR A655sc, Wilsonville, OR, USA) mounted on an observation port on the top of the build chamber.

The limited quantity of powder (~5 kg of each composition) prohibited an extensive experimental process parameter search. Instead, limits were imposed on the processing space based on our recent work on W [34] and our internal, preliminary work on pure Nb. Theoretically, Nb and W process parameters should represent the extremes of the processing space for these three alloys.

Two replicate builds of nine prismatic samples measuring 15 mm × 15 mm × 15 mm were fabricated for each composition. Among each of the nine samples, the beam speed was varied by adjusting the Arcam specific “speed function” (SF) parameter, the beam current (I) and beam focus offset current (FO) between predefined minimum and maximum value in increments of 5, 2, and 10, respectfully (SF = 5:5:20, I = 8:2:18 mA, FO = 10:10:50 mA). Additionally, for each composition, parameters were downselected from the prismatic sample experiments, based on relative density, to fabricate three tensile samples measuring 15 mm × 65 mm and two cylindrical samples measuring 15 mm in diameter. These downselected, tensile build parameters (Table 1) can be replicated by using the standard Arcam™ parameter set for Ti-6Al-4V (software version 3.2 SP2) and adjusting corresponding parameters to the values found in Table 1. Layer thickness was kept constant at 70 µm for each build.

Samples were fabricated until the powder in the deposition tank was exhausted; therefore, the sample height varied with the available powder quantity. The samples were oriented at an angle of 45 degrees about the machine *Z*-axis such that the X and Y-oriented scan paths resulted in identical scan line lengths and return times for the prismatic samples after hatch rotation. Build orientation is discussed further in Section 2.5. 

The samples were produced utilizing the standard control functionality wherein the CP-Ti build substrate was initially heated to 1250 °C prior to spreading the first layer. Subsequently, the surface temperature was maintained above 1000 °C (power analyze temperature) for the duration of the build using the preheating parameters in Table 1 over an 85 mm diameter area. A distinct advantage of the small build tank setup used in this study is heat distribution over a smaller volume, limiting heat transfer to surrounding components in the chamber which are stainless steel in most Arcam machines. This small build tank design has facilitated EB-PBF of other refractory materials including W [34], Mo+TiC [12], and C-103 [25].

### 2.3. Heat Treatment Scheduling

While it was intended that tungsten would be distributed to a nominal alloy composition within the liquid melt pool, the large gap in melting temperature between Nb and W, coupled with the relatively large W powder size, suggested that the peak temperature and time scales involved during EB-PBF would not be sufficient to fully dissolve the tungsten. Therefore, prior to tensile testing, 6 tensile bars from each composition were selected, at random, and subjected to a vacuum heat treatment at 1200 °C for 4 h and 12 h in a Thermo Scientific vacuum tube furnace under 5 × 10^−5^ mbar. Samples from the grip-ends of the bars were harvested and prepared for metallurgical analysis using the procedure described in Section 2.4. An additional heat treatment of 1200 °C for 165 h was conducted for particle dissolution studies, but these samples were not tensile tested.

An equation for the dissolution of spherical particles [35] was used to guide homogenization via heat treatment:

(1) $${R=R}_{o}-\frac{\mathrm{kDt}}{{2R}_{o}}-\frac{k}{\sqrt{\pi }}\sqrt{\mathrm{Dt}}\;$$

where R = dissolved particle radius, R_o_ = initial particle radius, D = interdiffusion coefficient, t = time, and

(2) $$k=\frac{{2(c}_{s}-{\;c}_{e})}{c_{c}-c_{s}}\;$$

**Figure 1:**
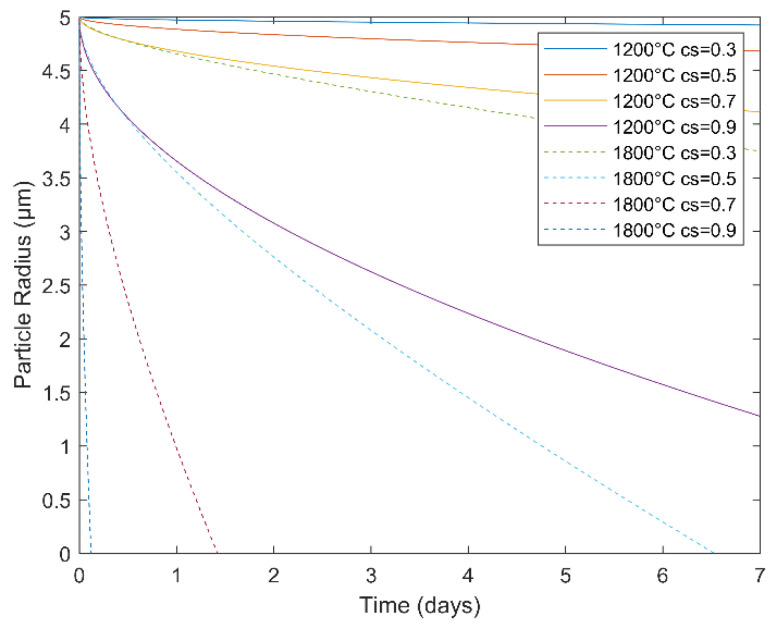
Dissolution kinetics model of spherical W inclusions in a Nb matrix during heat treatment of Nb-20W-1Zr-0.1C.

In determining the constant k, c_c_ = solute concentration in the precipitate, c_e_ = solute concentration in the matrix, and c_s_ = solute concentration at the interface. According to Road et al., logD = −14.5 at 1200 °C and −12.5 at 1800 °C for the interdiffusion of W inclusions in a Nb matrix [36]. The solute concentration was set to 100% in the precipitate and 10 or 20% in the matrix, depending on the alloy, and several interface concentration conditions were explored (30–90%). Figure 1 shows particle dissolution of a model W cluster (radius of 5 µm) in a Nb matrix as a function of heat treatment time for Nb-20W-1Zr-0.1C. This model not only serves as a simple predictive tool for heat treatment scheduling but also provides an estimate for the local concentration gradients surrounding W particles of a given size.

Optical analysis (ImageJ) of SEM images in backscatter mode was used to determine the initial average W particle radius in the fabricated samples and for the radius after each heat treatment condition.

### 2.4. Metallurgical Analysis

The cylindrical samples of the three compositions were longitudinally cross-sectioned on a LECO CM-10 saw with a diamond blade and prepared for metallurgical analysis by hot mounting and grinding 320–1200 grit, then polished using 1.0, 0.3, and 0.05 alumina slurry. Relative density was estimated using a contrast threshold optical technique from tiled, unetched optical micrographs and samples (ImageJ). This calculation did not include the first 2 mm of the samples, as these were intentionally made porous to prevent overmelting of the CP Ti build plate. Tiled images were generated using a Hirox KH-7700 digital optical microscope (Hirox-USA, Hackensack, NJ, USA). Microstructure and local composition of the samples were observed with SEM and EDS.

### 2.5. Mechanical Testing

**Figure 2:**
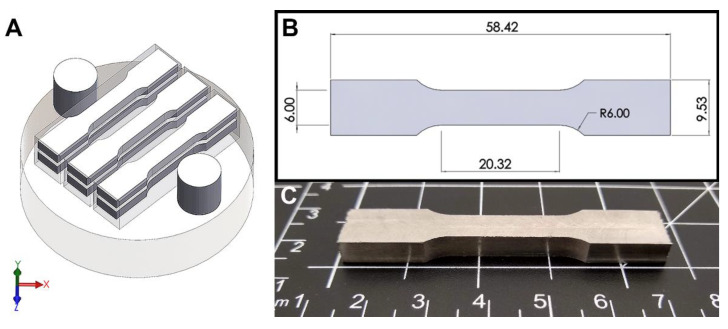
Illustration of the orientation and layout of samples on the CP-Ti substrate. (**A**) Tensile bar geometries and locations harvested by EDM are shown overlayed on the blocks that were produced. (**B**) Drawing showing the dimensions of the ASTM E8 Subsize tensile specimen and (**C**) a photograph of a finished tensile specimen. All dimensions in mm.

For each composition, modified ASTM E8 flat subsize tensile coupons were harvested from each of the 80 mm prismatic bars using wire electric discharge machining. 4 mm thick tensile bars were harvested with their gauge width (6 mm) parallel to the XY plane of the build (Figure 2). These subsize tensile bars were made shorter than the ASTM specs (32 mm gauge length, 30 mm grip length) so that 3 prismatic bars fit on the 90 mm build plate. Samples were cut slightly oversized and subsequently ground to remove any recast microstructure along the cut surface, which was later confirmed by SEM. The tests were performed using an ATS 1620C universal testing machine (Applied Test Systems, Butler, PA, USA) with a 100 kN load cell and a constant loading rate of 2.54 mm/min. Tensile samples were held in between a set of flat serrated wedge grips at the bottom and top, with the bottom end held static.

## 3. Results and Discussion

### 3.1. Powder Feedstock Characterization

**Figure 3:**
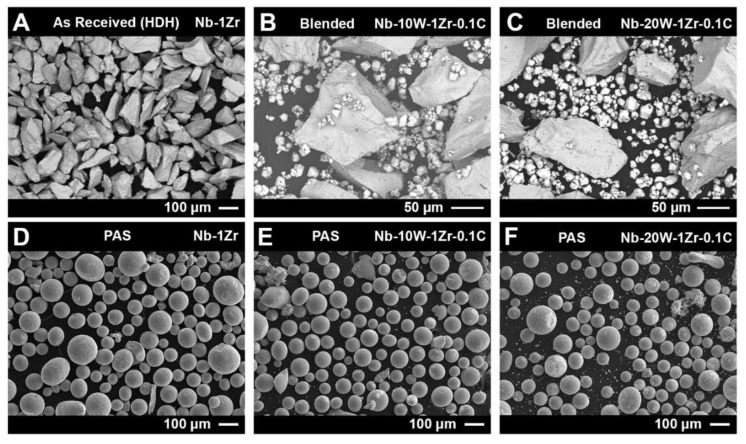
SEM images of as received (**A**) hydride-dehydride Nb-1Zr, (**B**) blended Nb-10W-1Zr-0.1C, (**C**) blended Nb-20W-1Zr-0.1C, and plasma alloy spheroidized (**D**) Nb-1Zr, (**E**) Nb-10W-1Zr-0.1C, and (**F**) Nb-20W-1Zr-0.1C.

Feedstock powder physical and chemical properties are known to have a significant influence on EB-PBF processing. Here, a 15–45 µm distribution of W particles was added to a larger distribution of HDH Nb-1Zr powder (45–106 µm) at 10 wt% W and 20 wt% W. Figure 3 shows the HDH powder was angular and faceted, as expected, and the fine W powder was acicular with large satellite particles. Following PAS, a large fraction of angular HDH Nb-1Zr was rounded to a comparable degree for each of the 3 materials. Few satellite particles and non-spherical morphologies were observed in Nb-10W-1Zr-0.1C but appeared more frequently in Nb-20W-1Zr-0.1C. Powders were not screened after initial blending to maintain nominal alloy compositions. This means that residual fine or ultrafine W particles, a bi-product of spheroidization, remained in the feedstock during EB-PBF.

The chemistry for each powder sample after PAS can be found in Table 2. As can be seen, significant losses of both W and C occurred. This is likely due to an inhomogenous distribution of fine W and carbon black powder in coarse Nb-1Zr, resulting in segregated regions of fine W powder (Figure 3B,C). Because the plasma flame had to be sufficiently hot to remelt the coarse Nb-1Zr powder, segregated W and C particles without Nb-1Zr as a nearest neighbor were evaporated. Non-spheroidized, as received (AR) W powder was added to both Nb-W PAS powders prior to EB-PBF to maintain nominal alloy values.

**Figure 4:**
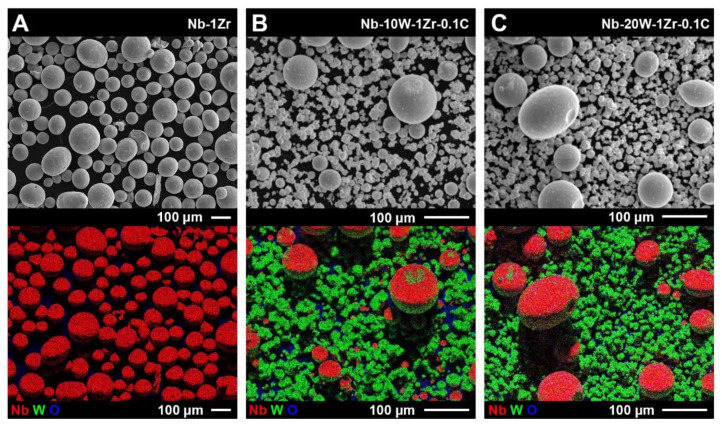
SEM and EDS composite RGB images of (**A**) spheroidized Nb-1Zr powder, (**B**) spheroidized and blended Nb-10W-1Zr-0.1C, and (**C**) spheroidized and blended Nb-20W-1Zr-0.1C.

SEM/EDS images of all three powders are shown in Figure 4. The nominal Nb-1Zr started as HDH powder and was mechanically crushed after hydride conversion. Nb-1Zr after PAS contained few detectable fine particles, though the morphology was not uniformly spherical. For the Nb-W powders, segregation resulted in a lack of intimate contact between the alloy constituents and only small regions of W are visible on the surface of the Nb-1Zr powder (Figure 4B,C). Alloying along the surface occurred during PAS, not during AR W reblend to return to nominal values, because 3D mixing involves less mechanical attrition than other common mixing techniques such as ball milling.

**Figure 5:**
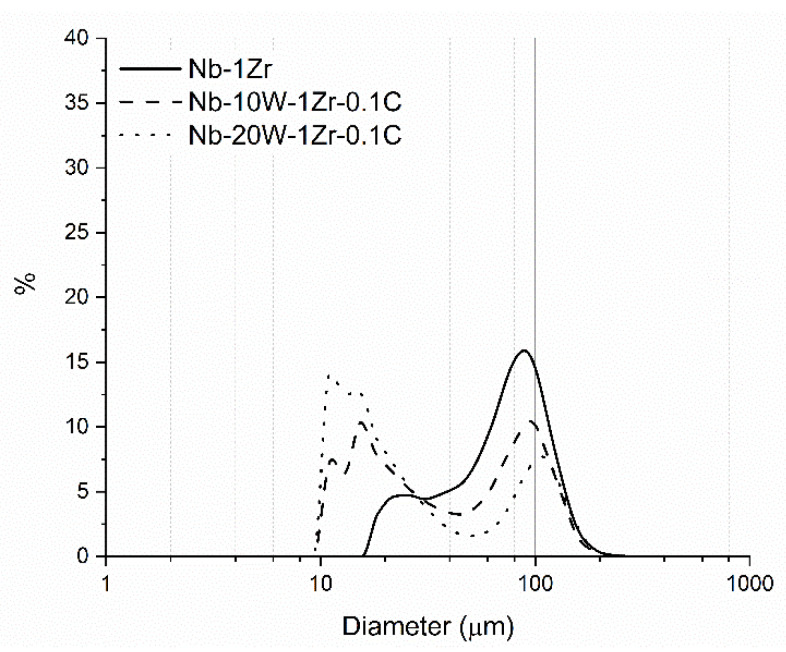
Numerical powder size distribution of the Nb-1Zr and blended Nb-W-Zr-C bimodal powders.

The acicularity of spheroidized Nb-1Zr manifested as an apparent “fines” content in the particle size distribution (PSD) between 20 and 30 µm, as shown in Figure 5. Despite these fine powders, relative to the high fraction of coarse powder in the distribution, the powder is considered typical of that used for EB-PBF processes. The fines content for the Nb-W alloys increases with increasing W content because of the reblend of AR W powder after PAS. This powder could not be screened to maintain nominal alloy composition and resulted in a bimodal distribution.

Bimodal powder distributions, characterized by higher powder bed packing fractions, can be amenable to EB-PBF processing for alloy constituents that have large melting point gaps. The melting point of tungsten is >1000 °C higher than that of Nb-1Zr, so the input EB-PBF parameters must provide sufficient energy to melt and solutionize the W while simultaneously avoiding overmelting of the Nb-1Zr. Incorporating smaller W particles than Nb-1Zr particles theoretically helps to bridge the melting point gap, because as particle size decreases, the surface area/volume ratio increases and less energy for melting is required as a result. This can also explain why segregated W powder was evaporated during PAS. A closely packed powder bed further enhances heat transfer between particles due to elimination of vacuum gaps. The degree of packing can be estimated by the Hausner ratio, which normalizes tap density by the bulk density of the powder. In this case, the Hausner ratio varied between 1.054 (Nb-1Zr) and 1.063 (Nb-20W-1Zr-0.1) which is not an appreciable difference. A Hausner ratio <1.25 is indicative of good flow and, by extension, spreadability.

### 3.2. Processing Space and Parameter Effects on Microstructure

**Figure 6:**
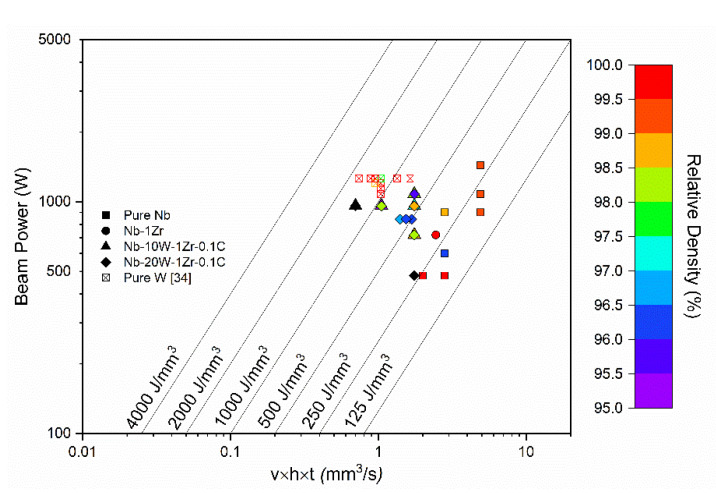
Process map for Nb-W alloys in comparison to pure Nb and pure W.

The melting point gap was found to have the most dominant effect on processing, despite the theoretical advantages of bimodal distributions. This can be seen explicitly in the process map included in Figure 6. The beam power is plotted against the beam speed (v, mm/s), the hatch offset (h, mm), and the vertical displacement of the build platform (t, mm). Lines of constant volumetric energy density (VED, J/mm^3^) are plotted diagonally. High-relative-density (>99%) samples of pure Nb and Nb-1Zr were formed between VED = 175–300 J/mm^3^, especially when beam power exceeded 600 W. Nb-10W-1Zr-0.1C and Nb-20W-1Zr-0.1C were highly dense at 300 J/mm^3^ but decreased sharply as VED approached 1500 J/mm^3^. VED of this magnitude is similar to that required for pure W, according to our EB-PBF and LPBF process maps [34]. The parameters meant to sufficiently melt the tungsten actually overmelted the Nb-1Zr matrix, leaving clusters of semi-melted W solidified in a defect-prone Nb-1Zr matrix.

**Figure 7:**
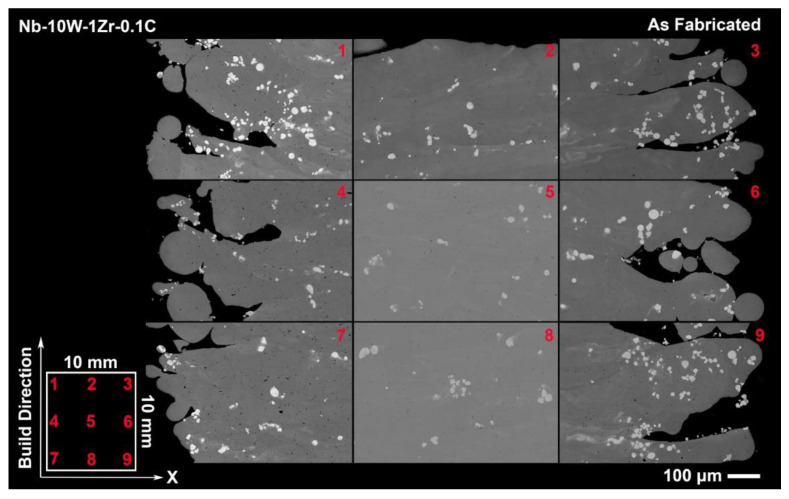
Tiled SEM image of as fabricated Nb-10W-1Zr-0.1C tensile sample witness cylinders depicting spatial microstructural variations.

**Figure 8:**
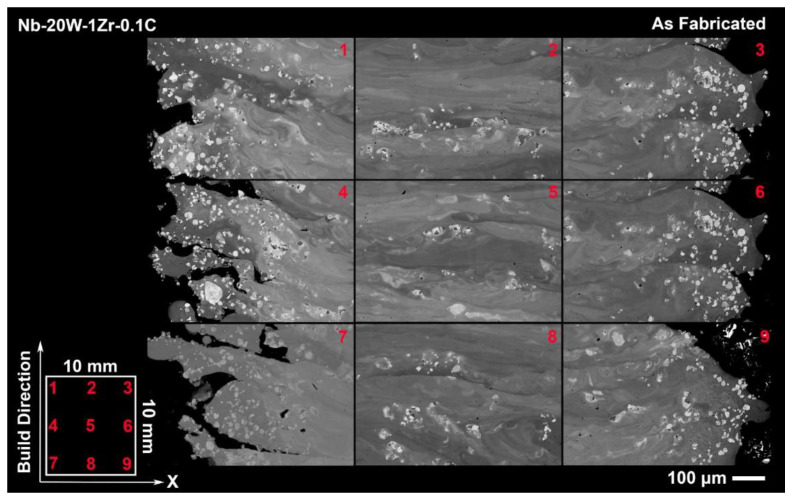
Tiled backscatter SEM image of as fabricated Nb-20W-1Zr-0.1C tensile sample witness cylinders depicting spatial microstructural variations.

SEM backscatter images of the as fabricated Nb-W alloys in Figure 7 and Figure 8 offer clear examples of the processing tradeoff within even a single sample and also as a function of W content. A combination of unmelted W particles and micropores can be seen in each of the 9 targeted sample locations along the build direction and across the sample’s length. Hazy films of dissolved W in the melt plane (XY) appear faintly in Nb-10W-1Zr-0.1C but become vividly defined in Nb-20W-0.1C, a contrast that is intuitive based on W fraction in each material. The films are approximately equal to the layer thickness (70 µm) in Figure 8 and repeat along the build direction (BD) at the center of the sample and along the edges. Partially melted W regions can be found in these films appearing more frequently along the edges in both alloys. Collectively, this suggests that possibly some W particles are carried by the melt pool and are deposited at the turning point.

It is important to note that while EB-PBF operates with a higher ambient temperature than other AM techniques, such as L-PBF, the powder bed temperature as measured by the top mounted pyrometer did not reach the levels required for W dissolution in this study. The powder bed was seen to fluctuate between 1000 and 1200 °C within a single layer, which would necessitate several days for appreciable dissolution, according to Figure 1. Post-processing heat treatments are discussed in subsequent sections.

**Figure 9:**
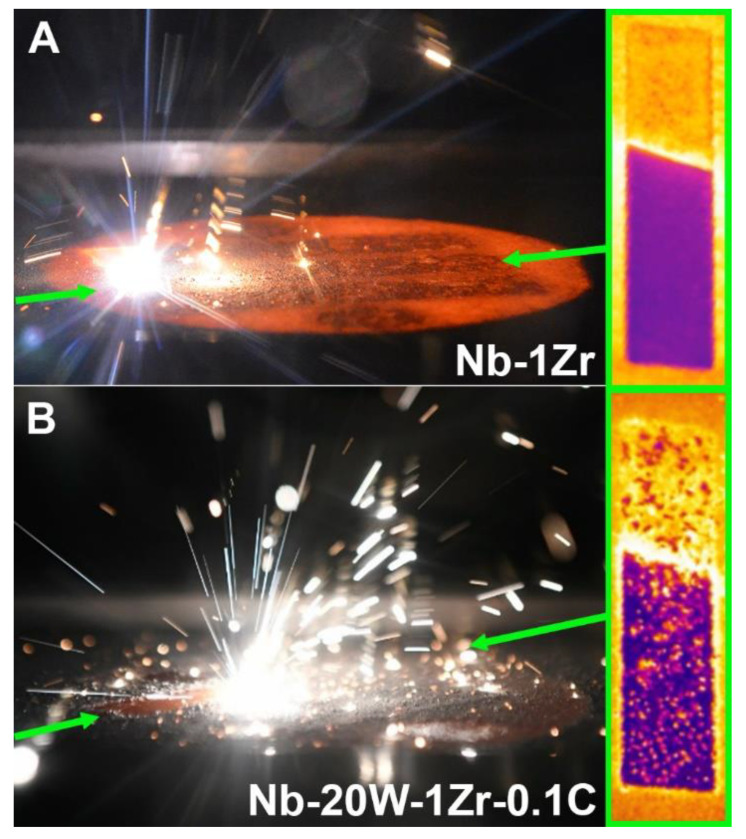
Build photos and IR images depicting (**A**) limited melt pool ejection in Nb-1Zr and (**B**) W cluster melt pool ejection typical of the Nb-W powders. IR images are not emissivity corrected and were taken from above during EB-PBF.

Fine W powders develop thick oxide shells that limit their ability to discharge electrons. When the charge accumulates and eventually exceeds a certain threshold, the powder is physically stimulated via electrostatic repulsion. This can manifest as melt pool ejection of W particle or particle clusters, which was commonly observed in this study for the Nb-W powders but not for Nb-1Zr (Figure 9). Ejected powder landed throughout the chamber, though several bright particles also landed on the surface of Nb-20W-1Zr-0.1C melt geometries as shown in the IR image included in Figure 9B. The IR images are not emissivity corrected to highlight the surface spatter. Taken together, with the fact that W particles were only marginally dissolved during processing and formed bands on the layer thickness scale, it stands to reason that tungsten clusters were actively moving in the melt pool, though further studies are required to verify this.

The combination of a layered compositional gradient along the build direction and observation of significant particle ejection, or spatter, during the melt steps suggests that that there are multiple sources of concentration gradient here. Table 3 shows that W content was not consistent with initial specifications after fabrication, despite the W reblend step that occurred after PAS but before EB-PBF. Because of the textured distribution of W clusters, especially in Nb-20W-1Zr-0.1C, and observed spatter, local composition gradients likely influenced the composition of samples harvested for chemical analysis and skewed the results, although they are within range of the feedstock powder chemistry in Table 2.

### 3.3. Heat Treatments and Diffusion

**Figure 10:**
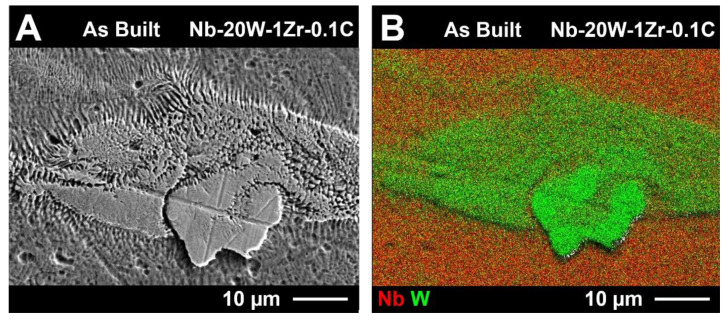
As-built Nb-20W-1Zr-0.1C (**A**) secondary SEM image and (**B**) EDS RGB composite image showing a graded composition and dendritic structure on the periphery of a tungsten particle.

It is interesting to note that W clusters were commonly accompanied by a local concentration gradient and that dendrites can also be found along the periphery of the W particles in the as-built condition (Figure 10). Similar behavior has been observed for W-Mo powder by Tan et al. [37]. Their group achieved fully dense, completely homogenized W-Mo powder after plasma spheroidization of agglomerated blended W and Mo powders. The W-Mo system is comparable to Nb-W in that both systems have total solid solubility between constituents; however, the melting point gap is much smaller between W and Mo than it is between Nb and W. Short dendrites of W in the as-built state indicate that limited diffusion occurred during fabrication, suggesting that complete homogenization can be attained with melting point gap mitigation. This limited diffusion has previously been reported by Prokoshkin et al. who found that W concentration decreases rapidly along the gradient at the Nb/W interface after heat treatment of 100 h at 1300 °C [38].

**Figure 11:**
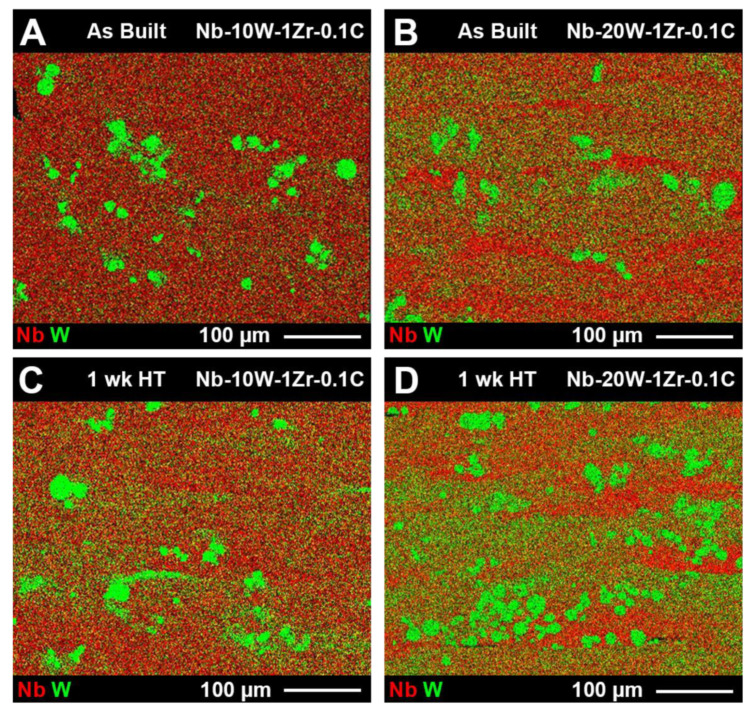
EDS RGB maps of (**A**) Nb-10W-1Zr-0.1C as-built, (**B**) Nb-20W-1Zr-0.1C as-built, (**C**) Nb-10W-1Zr-0.1C heat treated, and (**D**) Nb-20W-1Zr-0.1C heat-treated samples. Images were taken randomly near the center of the tensile build witness cylinders and are oriented in the same fashion as Figure 7 and Figure 8, region 5.

Discrete areas of elemental W measuring 10.0 ± 5.8 µm in diameter, on average, could be found in the as fabricated Nb-W materials. This diameter coincides directly with the lower limit of the Nb-W PSD’s in Figure 5, confirming that limited W melting occurred during EB-PBF. Heat treatments were performed for 4, 12, and 165 h at 1200 °C to further dissolve segregated W and to relieve residual stress. No discernible changes in particle size or W film intensity were evident for shorter heat treatments and the longer 1 week heat treatment had a marginal effect at this temperature (Figure 11). Although average W particle size was essentially the same as before heat treatment (10.8 ± 6.2 µm), there is a noticeable increase in the matrix W concentration after heat treatment for both Nb-W alloys.

### 3.4. Mechanical Properties

**Figure 12:**
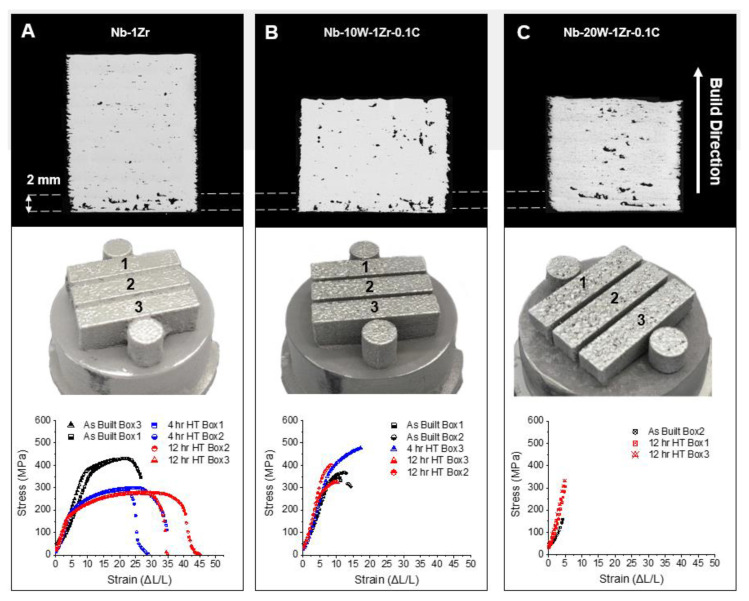
Optical micrographs of tensile build witness cylinders, tensile build photos, and corresponding properties for (**A**) Nb-1Zr, (**B**) Nb-10W-1Zr-0.1C, and (**C**) Nb-20W-1Zr-0.1C builds in the as-built and heat-treated (1200 °C) conditions.

Nb-1Zr attained the highest relative density (>99%) of the materials in this study because of the lack of spatter and fine W powder, meaning that its processing window was broader than the Nb-W alloys. As such, its microstructure had less of an effect on the tensile tests. The Nb-1Zr tensile properties shown in Figure 12A were significantly influenced by the high-temperature heat treatment where the yield decreased to a value of 157 MPa, UTS to 278 MPa, and elongation to 43%. Residual stresses incurred by the dynamic thermal environment, and also by the different thermophysical properties of Nb and Zr further contributed to inflated strengths and limited ductility in the as-built condition, which are known to revert to nominal levels after heat treatment [2,39].

The W-rich alloys contained more porosity and defects than Nb-1Zr due to the narrow processing window and complications in the build process, such as the spatter ejection and melting point gap. Their mechanical properties were inferior to traditional as a result. It can be seen in Figure 12B,C that the alloys failed prematurely during testing in most of the samples due to porosity, unmelted W-rich areas, and compositional gradients along the build direction. However, one of the Nb-10W-1Zr-0.1C samples yielded at 367 MPa and had a UTS of 468 MPa (Table 4) with an elongation of approximately 20%–values more representative of the traditional properties [40]. The same defects in Nb-10W-1Zr-0.1C samples were more pronounced in Nb-20W-1Zr-0.1C, leading to premature failure–although it should be noted that the 12 h heat treatment significantly improved the UTS through enhanced W cluster dissolution. While this improvement is encouraging, a more aggressive heat treatment schedule is likely required to fully dissolve the W and therefore realize traditional Nb-20W-1Zr-0.1C values.

## 4. Discussion

While the overall EB-PBF processability of blended Nb-1Zr and W powders has been demonstrated, there is substantial room for further studies to be conducted. PAS was intended to pre-alloy the powder, such that less energy would be required during EB-PBF and the processing window would broaden as a result. This would also reduce the heat treatment efforts in post-processing. Heat treatment experiments were designed to be viable by use of commercial furnaces. In lieu of non-segregated W powder, high-temperature furnaces capable of at least 1800 °C would be required to adequately dissolve the W in a reasonable amount of time; 50% in 6 days, according to Figure 1. Segregated W particles were not in close enough contact with Nb-1Zr to alloy during PAS. W coated Nb-1Zr powder is a possible solution to this problem, though additional preparations and costs would be incurred. Furthermore, PAS would still be required to remelt W oxide shells into the powders, else spatter susceptibility will remain an issue.

There also exists an opportunity to explore the sintering degree of the powder bed after preheating. Loosely sintered particles can disrupt heat and charge transfer which would mean W has a shorter path for interlayer diffusion after the beam passes. Shorting the powder bed’s circuit with loose powder can also cause the Arcam machine to arc trip, a challenging failure mechanism that can occur at any time during these builds.

Most importantly, loosely sintered powder is more likely to be ejected from the melt pool. Because of the large melting point gap (T_sinter_ = 0.6 T_m_), processing window limitations similar to that described for the melt parameters also exist for the preheat parameters that are responsible for sintering. Increasing the preheat energy input to ensure sintering of the W powder (T_sinter_ = 2053 °C) will result in oversintering of the Nb-1Zr powder (T_sinter_ = 1440 °C). Oversintered powder layers undergo a large thermal gradient that spans from the top surface (exposed to the beam) to the layers of powder below, resulting in uneven thermal expansion of the top layer that then separates from the powder cake. This is often referred to as “flaking”. Any material that protrudes or flakes from the build surface can be bumped and dragged by the raking mechanism, but it is especially catastrophic when this displaced material is part of the sinter cake. It is possible that a preheat process map as a function of sintering degree could advise preheat parameter selection to prevent both W spatter and oversintering.

Lastly, the optical analysis of unmelted particles discussed in this study was prone to errors and resulted in uncertainties of +50%. This partially explains why a difference in particle size before and after heat treatment could not be discerned, although an increase in matrix W concentration was observed. Alterative techniques such as x-ray computed tomography (CT) have been used to quantify unmelted Nb inclusions in a Ti-Nb alloy powder blend (Ti-34Nb) with a resolution of 5 µm [30] though commercial CT systems generally are not capable of penetrating a Nb-W alloy with satisfactory voxel resolution (<10 µm).

## 5. Conclusions

The research presented in this study demonstrates that highly dense Nb-1Zr, Nb-10W-1Zr-0.1C, and Nb-20W-1Zr-0.1C particles can be fabricated through EB-PBF via bimodal dissimilar powder blending followed by spheroidization. W powder segregation in the feedstock prevented effective alloying with Nb-1Zr, ultimately leading to unmelted W regions in both the as-built and heat-treated samples. It was shown that, despite the microstructural inhomogeneities, randomly distributed clusters of W suspended in a Nb-1Zr matrix can improve the yield strength and ultimate tensile strength of the virgin alloy while accompanied by an expected decrease in ductility. However, to realize mechanical properties of traditional high-strength Nb-W-Zr-C alloys, homogenization through Nb-W pre-alloying or particle coating should be explored. The melting point disparity between Nb-1Zr and W could not be alleviated by implementing bimodal powder distributions, nor via parameter compensation in the narrow processing window. Furthermore, the fine tungsten powder led to melt pool ejection and a layered composition gradient that increased in severity with increasing W content. Post-processing can mitigate these defects, but heat treatment schedules more aggressive than those used here are needed to maximize the effect. The authors consider that if powder feedstock alloying issues can be resolved, Nb materials of various compositions, with melting temperatures exceeding 2500 °C, can be EB-PBF melted to form dense, near-net shapes for high-temperature structural applications.

## Figures and Tables

**Table 1:** EB-PBF parameters specific to the Arcam control system that were used to fabricate the tensile samples in this study (variation from the standard commercial parameter set for Ti6Al4V).

Parameter	Nb-1Zr	Nb-10W-1Zr-0.1C	Nb-20W-1Zr-0.1C
Melt: Power Analyze (°C)	1300	1300	1300
Melt: Beam Speed (mm/s)	200	200	200
Melt: Current (mA)	12	12	14
Melt: Max Current (mA)	12	12	14
Melt: Focus Offset (mA)	35	35	50
Melt: Speed Function	13	13	10
Melt: Line Offset (mm)	0.175	0.125	0.11
Preheat: Square (mm)	80	80	80
Preheat 1: Line Order	20	20	20
Preheat 1: Line Offset (mm)	1.2	1.2	1.2
Preheat 1: Hatch Depth (mm)	0.1	0.1	0.1
Preheat 1: Max Beam Current (mA)	32	32	32
Preheat 1: Min Beam Current (mA)	5	5	5
Preheat 1: Beam Speed (m/s)	10	10	10
Preheat 1: Number of Repetitions	30	30	45
Preheat 1: Max Number of Repetitions	30	30	45
Preheat 1: Average Current (mA)	20	20	20

**Table 2:** Chemistry as measured by ICP-OES and LECO for Nb-1Zr as received powder, and for all 3 alloy powders after PAS and Nb-W-Zr-C powders.

	Nb-1Zr (AR)	Nb-1Zr (PAS)	Nb-10W-1Zr-0.1C (PAS)	Nb-20W-1Zr-0.1C (PAS)
Nb (wt%)	BAL	BAL	BAL	BAL
W (wt%)	0.0054 †	0.0056 †	1 †,*	2 †,*
C (wt%)	0.009	0.0083	0.034	0.032
O (ppm)	830	1000	870	990

† = ICP-OES. * manually adjusted to nominal before EB-PBF.

**Table 3:** Chemical analysis of solid EB-PBF fabricated samples.

	Nb-1Zr	Nb-10W-1Zr-0.1C	Nb-20W-1Zr-0.1C
Nb (wt%)	BAL	BAL	BAL
W (wt%)	0.047 †	13.2 †	25.1 †
Zr (wt%)	0.85 †	0.73 †	0.64 †
C (wt%)	0.012	0.024	0.022
O (ppm)	974	851	971
H (ppm)	41.5	20.4	21.5

† = ICP-OES.

**Table 4:** Tensile properties of as fabricated and heat-treated Nb-1Zr, Nb-10W-1Zr-0.1C, and Nb-20W-1Zr-0.1C.

Material	Heat Treatment	Yield Strength (MPa)	Ultimate Tensile Strength (MPa)	Strain (∆L/L)
Nb-1Zr	As Built	366.5 ± 0.5	428.5 ± 2.0	23.6 ± 3.3
Nb-1Zr	4 h 1200 °C	192.5 ± 0.5	295.6 ± 4.4	32.0 ± 2.9
Nb-1Zr	12 h 1200 °C	157.5 ± 9.5	277.6 ± 2.5	40.1 ± 5.2
Nb-10W-1Zr-0.1C	As Built	301.0 ± 4.0	357.3 ± 11.2	13.2 ± 1.6
Nb-10W-1Zr-0.1C	4 h 1200 °C	367.0 ± 0.0	478.2 ± 0.0	17.9 ± 0.0
Nb-10W-1Zr-0.1C	12 h 1200 °C	290.0 ± 45.0	367.2 ± 37.7	10.3 ± 1.4
Nb-20W-1Zr-0.1C	As Built	-- ± --	170.6 ± N/A	4.3 ± N/A
Nb-20W-1Zr-0.1C	12 h 1200 °C	-- ± --	327.8 ± 17.7	5.0 ± 0.1

## Data Availability

Data is contained within the article.
